# A non-diploid DNA status is linked to poor prognosis in renal cell cancer

**DOI:** 10.1007/s00345-020-03226-8

**Published:** 2020-05-02

**Authors:** Franziska Büscheck, Christoph Fraune, Martina Kluth, Maximilian Lennartz, Ronald Simon, Claudia Hube-Magg, Christian Morlock, Silvano Barbieri, Carolin Wahl, Christian Eichelberg, Christina Möller-Koop, Doris Höflmayer, Corinna Wittmer, Waldemar Wilczak, Guido Sauter, Margit Fisch, Till Eichenauer, Michael Rink

**Affiliations:** 1 Institute of Pathology, University Medical Center Hamburg-Eppendorf, Martinistr. 52, 20246 Hamburg, Germany; 2 Urologische Praxis Straubing, Stadtgraben 1, 94315 Straubing, Germany; 3 Department of Urology, University Medical Center Hamburg-Eppendorf, Martinistr. 52, 20246 Hamburg, Germany

**Keywords:** Renal cell cancer, DNA ploidy, Prognosis, Flow cytometry

## Abstract

**Purpose:**

DNA ploidy measurement has earlier been suggested as a potentially powerful prognostic tool in many cancer types, but the role in renal tumors is still unclear.

**Methods:**

To clarify its prognostic impact, we analyzed the DNA content of 1320 kidney tumors, including clear cell, papillary and chromophobe renal cell carcinoma (RCC) as well as renal oncocytoma and compared these data with clinico-pathological parameters and patient prognosis.

**Results:**

A non-diploid DNA content was seen in 37% of 1276 analyzable renal tumors with a striking predominance in chromophobe carcinoma (74.3% of 70 cases). In clear cell carcinoma, a non-diploid DNA content was significantly linked to high-grade (ISUP, Fuhrman, Thoenes; *p* < 0.0001 each), advanced tumor stage (*p* = 0.0011), distant metastasis (*p* < 0.0001), shortened overall survival (*p* = 0.0010), and earlier recurrence (*p* < 0.0001). In papillary carcinoma, an aberrant DNA content was significantly linked to high Fuhrman grade (*p* = 0.0063), distant metastasis (*p* = 0.0138), shortened overall survival (*p* = 0.0010), and earlier recurrence (*p* = 0.0003).

**Conclusion:**

In summary, the results of our study identify a non-diploid DNA content as a predictor of an unfavorable prognosis in clear cell and papillary carcinoma.

**Electronic supplementary material:**

The online version of this article (10.1007/s00345-020-03226-8) contains supplementary material, which is available to authorized users.

## Introduction

Renal cell carcinoma (RCC) is the ninth most common malignant tumor worldwide [1]. Localized tumors are generally treated by nephrectomy or, whenever feasible, by partial nephrectomy. For patients requiring systemic therapy, several new drugs have been approved lately which have improved the still unfavorable prognosis of metastatic disease [2, 3]. It is currently evaluated in clinical trials, whether adjuvant treatment with new drugs can improve the prognosis of localized kidney cancer in patients at high risk for disease recurrence or progression after nephrectomy (Keynote-564, iMmotion 010, CheckMate 914). If adjuvant treatment becomes standard of care, risk stratification will become more important than ever before, to find out which patient is at risk and might benefit from adjuvant therapies.

**Fig. 1:**
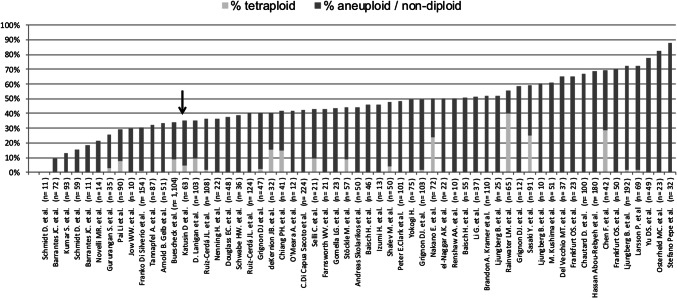
Literature overview

Measurement of the cellular DNA content by either flow cytometry or static image cytometry has been extensively discussed as a potential prognostic tool in many cancer types in the 1980s and early 1990s [4–9]. At that time, ploidy measurement represented the best possible method to global assess tumor DNA content. It was postulated that a non-diploid DNA status would indicate genomic instability, which in principle should be linked to an aggressive cancer phenotype. Various studies have demonstrated that tetraploidy and aneuploidy may be associated with unfavorable disease outcome in many cancer types, such as cancers of the colon, breast, oral cavity, urinary bladder and lungs [10–15]. Even though the method is inexpensive and yields highly reproducible data, cytometry has not become a routine tool in any of these cancers, partially because the prognostic information was clinically not relevant at the time when studies were performed. Studies evaluating DNA ploidy in renal neoplasm analyzed particularly small cohorts mostly below 100 patients and came to varying conclusions (Fig. 1, [16–74]). Some reports have suggested that a non-diploid DNA content may have substantial clinical importance in RCC [19, 26, 27, 32, 35, 45, 48, 51].

Given the increased need for prognostic markers in the era of adjuvant therapy, this study was to reassess the prognostic impact of aberrant DNA content in kidney tumors. For this purpose, a cohort of kidney tumors, including clear cell, papillary and chromophobe RCC as well as renal oncocytoma was analyzed by DNA flow cytometry.

## Materials and methods

### Patients

A set of 1320 consecutive kidney tumors surgically removed between 1994 and 2015 and where the tumor was histopathologically evaluated at the Institute of Pathology of the University Medical Center Hamburg-Eppendorf, Germany was used. The patients were treated according to the German and international guidelines. Localized tumors were treated by radical nephrectomy or partial nephrectomy, whenever feasible. In few metastatic patients, cytoreductive nephrectomy was performed. If systemic therapy was indicated, most of the patients received tyrosine kinase inhibitors, as recommended by the valid guidelines until recently. All tumors were reviewed according to the criteria described in the 2016 WHO classification by two pathologists with a special focus on genitourinary pathology (FB, CF). Fuhrman, Thoenes, and WHO/ISUP grading was performed for each tumor. Clinical and pathological parameters of the arrayed tumors are summarized in Table 1. The mean follow-up time was 41 months (range 1–250 months). Overall survival and progression-free survival data were collected retrospectively from patient records including data from attending physicians. Progression was defined as initial occurrence of metastasis in initial M0 patients or clinical progress in case of initial M1 patients. The usage of archived diagnostic left-over tissues for research purposes as well as patient data analysis has been approved by local laws (HmbKHG, §12) and by the local ethics committee (Ethics commission Hamburg, WF-049/09). All work has been carried out in compliance with the Helsinki Declaration.

**Table 1:** Study cohort

	Study cohort (*n* = 1320)
Follow-up	
Available (*n*)	867
Mean (months)	41
Median (months)	60
Age (years)	
< 50	196
50–70	729
70–90	395
Histology	
Clear cell RCC	831
Papillary RCC	206
Chromophobe RCC	73
Oncocytoma	125
UICC stage	
I	570
II	87
III	119
IV	111
pT category	
pT1	734
pT2	157
pT3–4	274
ISUP grade	
1	323
2	358
3	323
4	66
Fuhrman grade	
1	56
2	621
3	325
4	77
Thoenes grade	
1	380
2	597
3	102
pN category	
pN0	176
PN +	37
pM category	
pM0	174
pM +	106

Numbers do not always add up to 1320 in the different categories because of missing data

### Flow cytometry

Cell nuclei were extracted from formalin-fixed paraffin-embedded tissues and stained for flow cytometry analysis using a modified standard protocol [75–77]. In brief, two punches (diameter 0.6 mm) per tissue block were taken with a hollow needle from 1320 kidney neoplasm included in this study. For preparation of cell suspensions, punches were re-embedded in paraffin, and cut into 50 µm sections using a microtome. The sections were placed in a nylon bag with a mesh of 50 µm, dewaxed in xylene and rehydrated in a descending series of ethanol (100%, 96%, 70%, water) prior to digestion in 5 mg/ml pepsin (Serva #31,820.02, Heidelberg, Germany), and resolved in 0.07 M HCl in a 37 °C water bath for 30 min. 5 ml cold phosphate buffered saline was added to stop the reaction. The suspension was centrifuged at 2,500 rpm to release cell nuclei from the nylon bags, resuspended to a volume of 450 µl and transferred to 5 ml fluorescence-activated cell sorting.

### (FACS) tubes

RNAse A (Sigma #R4875, St. Louis, USA) was added to a final concentration of 0.05 mg/ml (adjusted to pH 7.4) before incubation at 37 °C for 30 min. Nuclei were then stained by adding 100 µl propidium iodide solution (1 mg/ml) (Sigma, #P4864) for 5 min at 4 °C in the dark. The DNA content was measured in at least 1000 stained nuclei using a FACS Canto II using the blue laser (488 nm) with the filter configuration longpass 556 nm and bandpass 585/42 nm.

### Interpretation of the FACS results

Results of the FACS analysis were evaluated as follows: exclusion of cell duplicates and propidium iodide negative particles, creation of FACS flow histogram, setting measurement range for each clearly visible peak, and determination of DNA content with the following standard criteria as described before [77–79]. The first analyzable peak, indicating the fraction of cells with the smallest DNA content, was considered DNA diploid and was given a DNA index of 1.0. DNA indices for the following peaks were calculated relative to the 1.0 peak. Samples were considered DNA aneuploid when one or more unequivocal peaks between DNA index 1.0 and < 1.8 or > 2.2 was present. Tetraploidy was defined as the presence of one unequivocal peak between DNA index 1.8 and 2.2 with more than 15% of all cells. Otherwise, the sample was considered DNA diploid.

### Statistics

Statistical calculations were performed with JMP 11 software (SAS Institute Inc., NC, USA). Contingency tables and the chi^2^ test were performed to search for associations between molecular parameters and tumor phenotype. Survival curves were calculated according to Kaplan–Meier. The log-rank test was applied to detect significant differences between groups.

## Results

### DNA ploidy and histological renal tumor subtypes

**Fig. 2:**
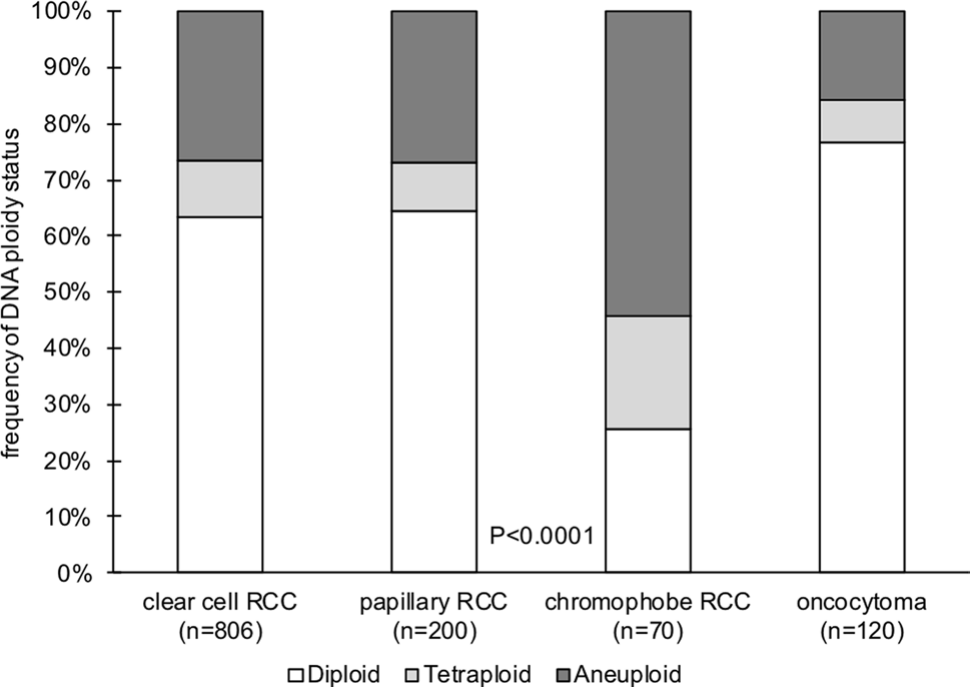
DNA ploidy status in histological subtypes of renal cell cancer and renal oncocytoma

1276 from 1320 tumors were interpretable (96.7%). Of these tumors, 802 (62.9%) were diploid, 127 (10.0%) were tetraploid, and 347 (27.2%) were aneuploid. Results for clear cell RCC (36.6% non-diploid cases) and papillary RCC (35.5% non-diploid cases) were similar, but markedly higher frequencies of non-diploid cancers were found in chromophobe RCC (74.3%, *p* < 0.0001 vs clear cell carcinoma). Results for carcinoma subtypes and renal oncocytoma are shown in Fig. 2.

### DNA ploidy and tumor phenotype

**Fig. 3:**
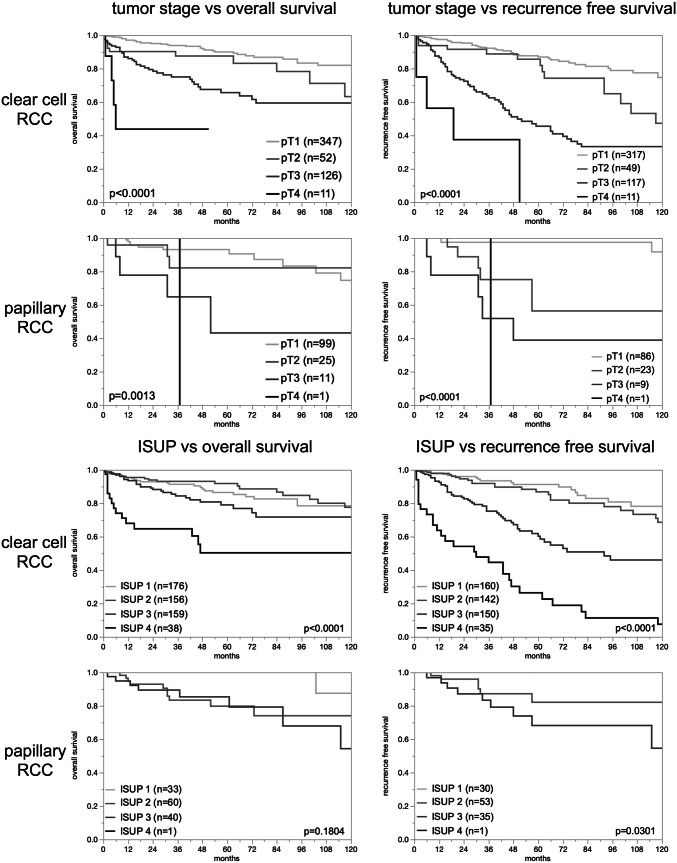
Prognostic impact of tumor stage and ISUP

**Fig. 4:**
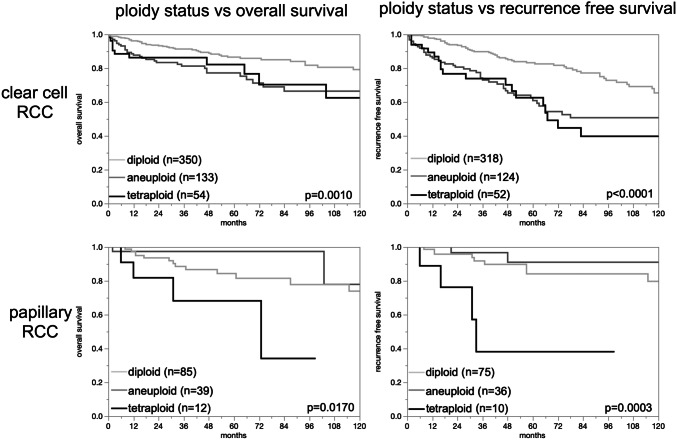
Prognostic impact of the DNA ploidy status

Given the marked ploidy differences between renal tumor subtypes, the relationship with tumor phenotype and patient outcome was separately analyzed in clear cell and papillary RCC subtype (Table 2). In clear cell RCC, the percentage of tumors with a non-diploid DNA content raised significantly with adverse tumor features, including high-grade (ISUP, Fuhrman, Thoenes, *p* < 0.0001 each), high UICC stage (*p* < 0.0001), advanced pT stage (*p* = 0.0011), and presence of distant metastasis (*p* < 0.0001). In papillary RCC, a non-diploid status was only significantly linked with high Fuhrman grade (*p* = 0.0063) and the presence of distant metastasis (*p* = 0.0138; *p* ≤ 0.0001 if aneuploid and tetraploid was combined). The validity of the follow-up data was documented by a striking prognostic impact of the pT category and ISUP in clear cell RCC and papillary RCC (*p* ≤ 0.05 except for ISUP in papillary carcinoma, Fig. 3). Univariate analysis showed that a non-diploid DNA status was linked to reduced overall survival in 537 clear cell (*p* = 0.0010) and 136 papillary RCC (*p* = 0.0170) with available follow-up data. In both subgroups, a non-diploid DNA status was also associated with reduced recurrence-free survival (*p* ≤ 0.0003; Fig. 4). Multivariate analysis showed no additional impact of the ploidy status beyond tumor stage, tumor grade or status of metastasis for overall survival and recurrence-free survival (Supplementary Table 1).

**Table 2:** DNA ploidy status and tumor phenotype

Parameter	Clear cell renal cell carcinoma					Papillary renal cell carcinoma				
	*n*	Diploid	Tetraploid	Aneuploid	*p* values	*n*	Diploid	Tetraploid	Aneuploid	*p* values
ISUP										
1	256	83.6	4.3	12.1	< 0.0001	42	64.3	9.5	26.2	0.1666
2	245	63.7	9.4	26.9		90	74.4	5.6	20.0	
3	242	47.5	15.3	37.2		65	52.3	10.8	36.9	
4	54	37.0	16.7	46.3		1	100.0	0.0	0.0	
Fuhrman										
1	49	81.6	2.0	16.3	< 0.0001	2	100.0	0.0	0.0	0.0063
2	448	73.9	6.7	19.4		131	73.3	6.9	19.9	
3	247	47.0	15.4	37.7		62	46.8	9.7	43.6	
4	61	39.3	19.7	41.0		3	66.7	33.3	0.0	
Thoenes										
1	301	82.7	4.0	13.3	< 0.0001	51	66.7	9.8	23.5	0.1123
2	425	55.3	11.1	33.7		139	64.0	6.5	29.5	
3	79	34.2	27.9	38.0		8	75.0	25.0	0.0	
UICC										
1	381	69.0	8.1	22.8	< 0.0001	110	68.2	6.4	25.5	0.1971
2	50	68.0	8.0	24.0		22	68.2	9.1	22.7	
3	98	55.1	15.3	29.6		6	50.0	0.0	50.0	
4	82	35.4	12.2	52.4		13	53.9	30.8	15.4	
Tumor stage (pT)										
1	484	68.4	8.1	23.6	0.0011	140	65.7	6.4	27.9	0.4260
2	94	64.9	10.6	24.5		40	65.0	15.0	20.0	
3	211	51.7	14.7	33.7		11	54.6	18.2	27.3	
4	11	36.4	9.1	54.6		2	100.0	0.0	0.0	
Lymph node metastasis (pN)										
pN0	18	38.9	11.1	50.0	0.1939	8	50.0	37.5	12.5	0.1211
pN +	134	57.5	14.2	28.4		18	83.3	5.6	11.1	
Distant metastasis (pM)										
pM0	118	68.6	8.5	22.9	< 0.0001	26	84.6	0.0	15.4	0.0138
pM1	84	35.7	14.3	50.0		6	66.7	33.3	0.0

## Discussion

A total of 37% of 1276 interpretable kidney tumors were non-diploid in this study. This frequency lies within the lower and the middle third of the published rate of ploidity in RCC (Fig. 1). Earlier studies on 10–224 RCC reported frequencies of non-diploid cases between 10 and 70% [16–74]. The five studies describing even higher aneuploidy rates (i.e. > 70%) had employed more sensitive procedures such as analyzing multiple samples per tumor [17, 19, 73], using ultrasensitive (and less specific) definitions of aneuploidy [23], or analyzed special cancer types such as Wilms tumors [21].

The comparison of different kidney tumor entities identified chromophobe carcinoma as unique with respect to its DNA content. That aneuploidy and tetraploidy is twice as frequent than in other major subtypes fits well with earlier cytogenetic data describing chromophobe carcinoma as a tumor with numerous chromosomal imbalances [18]. It is of note, that chromophobe kidney cancer is a tumor with a rather good prognosis [80–82]. Therefore, the high prevalence of aneuploidy in chromophobe kidney cancer conflicts with the general concept of non-diploidy representing a feature of poor clinical outcome. It is also noteworthy that non-diploid cases were seen in more than 15% of cases of renal oncocytomas, which is the benign counterpart of chromophobe RCC. This demonstrates that a non-diploid DNA content does not define malignancy. Aneuploidy has also been reported in other benign lesions, such as follicular adenomas of the thyroid [83, 84]. In agreement with our data, non-diploid DNA status was reported in six of seven chromophobe kidney carcinomas and in one of six oncocytomas in one earlier study [18].

In most cancer types, the fraction of non-diploid cases increases with dedifferentiation, i.e. high tumor grade [85–87]. In this study, such an association was found for the newly introduced WHO/ISUP grade as well as for the older Fuhrman and Thoenes grades. Several earlier studies have reported similar data [16, 20, 22]. The large number of morphologically well-characterized kidney tumors collected for this study allows a tumor subtype-specific comparison of DNA content with morphological tumor characteristics and clinical outcome. The outcome analysis of ploidity in clear cell RCC—the largest tumor type subgroup with available follow-up data of 537 tumors—revealed an adverse correlation with survival. However, this association was less strong compared to the prognostic effect of the established grading systems, including WHO/ISUP. Together with the high aneuploidy rate in the rather indolent chromophobe RCC, this illustrates well that ploidy status is a biologically interesting tumor phenomenon probably linked to certain type of genomic instability but not a key determinant of tumor aggressiveness. In addition, it is well known that chromophobe cancers are characterize by losses of gross chromosomal material [88] which might contribute to changes in the cellular DNA content. It is also well conceivable that the behavior of a cancer cell is more dependent on what specific pathways are activated or inactivated than on the total quantity of cellular DNA.

Given the predominance of clear cell subtype among kidney tumors, most studies investigating potentially relevant prognostic molecular features focus on clear cell RCC and lack larger cohorts on non-clear cell tumors that permit to draw reliable conclusions on the prognostic impact of biomarkers in non-clear cell subtypes. Although our study represents the largest of its kind, the subgroup of 200 papillary cancers is still rather small for statistical analyses. Accordingly, associations of tumor stage and the applied grading systems with clinical outcome were less clear than for clear cell carcinoma. That cytometry data lacked significant association with all analyzed clinico-pathological parameters in papillary RCC again illustrates the limitation of DNA content as a clinically meaningful prognosticator in kidney cancer.

In summary, our study clarifies the prognostic value of DNA ploidy changes in an unprecedented large cohort of more than 1000 kidney tumors. A high DNA content was linked to unfavorable clinical outcome in univariate analysis; however, it did not provide additional prognostic information beyond established prognostic parameters including the new WHO/ISUP grading. The strikingly higher rate of aneuploidy in chromophobe RCC than in other kidney tumors provides further arguments for this tumor to represent a biologically distinct tumor entity potentially responding differently to treatments. Chromophobe RCC needs to be reliably distinguished from clear cell carcinoma by pathologists.

## Electronic supplementary material

Below is the link to the electronic supplementary material.

Supplementary file1 (XLSX 9 kb)

## Abbreviations

FACS: Fluorescence-activated cell sorting
RCC: Renal cell carcinoma
